# Evaluation of photobiomodulation therapy associated with guided bone regeneration in critical size defects. *In vivo* study

**DOI:** 10.1590/1678-7757-2017-0244

**Published:** 2018-04-18

**Authors:** Nicole Rosa de Freitas, Luísa Belluco Guerrini, Luis Augusto Esper, Michyele Cristhiane Sbrana, Gisele da Silva Dalben, Simone Soares, Ana Lúcia Pompéia Fraga de Almeida

**Affiliations:** 1 Universidade de São Paulo, Faculdade de Odontologia de Bauru, Pós-Graduação em Reabilitação Oral, Bauru, São Paulo, Brasil; 2 Universidade de São Paulo, Hospital de Reabilitação de Anomalias Craniofaciais, Seção de Periodontia, Bauru, São Paulo, Brasil; 3 Universidade de São Paulo, Hospital de Reabilitação de Anomalias Craniofaciais, Seção de Odontopediatria e Saúde Coletiva, Bauru, São Paulo, Brasil; 4 Universidade de São Paulo, Faculdade de Odontologia de Bauru, Departamento de Prótese e Periodontia, Bauru, São Paulo, Brasil

**Keywords:** Autograft, Bone regeneration, Lasers

## Abstract

The repair of bone defects raises the interest of investigators in several health specialties. Grafting techniques with bone substitutes and laser therapies have been investigated to replace autogenous bone and accelerate the bone healing process.

**Objective:**

To evaluate the effect of photobiomodulation therapy (PBMT) associated with guided bone regeneration (GBR) in critical size defects.

**Material and Methods:**

The study was conducted on 80 male rats (*Rattus norvegicus albinus,* Wistar) submitted to surgical creation of a critical size defect on the calvaria, divided into eight study groups: group C (control - only blood clot); group M (collagen membrane); group PBMT (photobiomodulation therapy); group AB (autogenous bone); group AB+PBMT; group AB+M; group PBMT+M; group AB+PBMT+M. The animals were killed 30 days postoperatively. After tissue processing, bone regeneration was evaluated by histomorphometric analysis and statistical analyses were performed (Tukey test, p<0.05).

**Results:**

All groups had greater area of newly formed bone compared to group C (9.96±4.49%). The group PBMT+M (achieved the greater quantity of new bone (64.09±7.62%), followed by groups PBMT (47.67±8.66%), M (47.43±15.73%), AB+PBMT (39.15±16.72%) and AB+PBMT+M (35.82±7.68%). After group C, the groups AB (25.10±16.59%) and AB+M (22.72±13.83%) had the smallest quantities of newly formed bone. The area of remaining particles did not have statistically significant difference between groups AB+M (14.93±8.92%) and AB+PBMT+M (14.76±6.58%).

**Conclusion:**

The PBMT utilization may be effective for bone repair, when associated with bone regeneration techniques.

## Introduction

The need to repair bone defects has raised the interest of investigators in several health specialties. Grafting techniques with bone substitutes and laser therapies^2^ ^,^ ^3^ ^,^ ^6^ ^,^ ^24^ have been investigated to replace autogenous bone and accelerate the bone healing process.

Autogenous bone is considered the gold standard for regeneration of bone defects^9^, because of its osteogenic, osteoinductive and osteoconductive properties^5^ ^,^ ^9^. Also, it does not have antigenicity^9^, but a natural supporting structure and type I collagen, which promotes resilience and revascularization^9^. However, its achievement is associated with postoperative complications at the donor site, such as esthetic defects, hematomas, injuries to local anatomic structures, infections, and postoperative pain^7^. Another aspect to be considered is the unpredictable graft resorption, which may negatively influence the postoperative outcomes.

Photobiomodulation therapy (PBMT) has been used in Dentistry in extraction sockets, bone fractures and postoperative period of surgeries. The laser promotes biostimulation or photobiomodulation^8^ ^,^ ^27^, modifying the cell behavior, changing the mitochondrial respiratory chain and calcium channels, possibly increasing the production of adenosine triphosphate (ATP), collagen synthesis and angiogenesis. Due to these fast cell divisions and proliferations, migration of fibroblasts and production of bone matrix^25^, the laser stimulates the repair of bone defects^24^ implanted with autogenous bone grafts^2^ ^,^ ^17^ ^,^ ^26^, biomaterials, as inorganic bone, lyophilized bone, hydroxyapatite, and morphogenetic bone protein, associated or not with biological membranes^6^ ^,^ ^16^.

The utilization of biological membranes in guided bone regeneration (GBR) prevents epithelial migration into the bone defect, besides maintaining space to allow the host osteogenic cells to repopulate the defect area and accelerate the bone repair^11^. The membranes should be biocompatible, able to create space, have occlusive properties, achieve tissue integration and be easy to handle.

The association of membrane with autogenous graft improves the outcomes, since its utilization stabilizes the grafting material inside the bone defect, which may enhance the outcomes achieved so far when associated with PBMT. Thus, this study evaluated the effect of PBMT associated with guided bone regeneration in critical size defects. The null hypothesis was that PBMT does not influence on the healing of critical size defects when associated with guided bone regeneration.

## Material and methods

This study was approved by the Institutional Review Board for Animal Studies of Bauru School of Dentistry, University of São Paulo (CEEPA- Proc N° 023/2014).

### Study design

The sample had 80 male rats *(Rattus norvegicus albinus,* Wistar), weighing 250 to 300 g, supplied by the Central Animal Laboratory of Bauru School of Dentistry, University of São Paulo. The animals were kept in an environment with 12-hour daylight cycle and temperature between 22 and 24°C and received selected solid food and water *ad libitum.* They were randomly divided into groups by drawing lots containing the group number to which they should belong, which was performed by an investigator not participating in surgical procedures and unaware of which treatment would be performed, thus “blinded” to the experiment. The animals were divided into eight study groups (n = 10): group C (control - only blood clot); group M (collagen membrane - BioGide^®^, Geistlich Pharma AG, Wolhusen, Switzerland); group PBMT (low-level laser - TheraLase DMC^®^, DMC Equipamentos Ltda., São Carlos, SP, Brazil); group AB (autogenous bone); group AB+PBMT; group AB+M; group PBMT+M; group AB+PBMT+M.

### Surgical procedure

A single operator performed the surgical procedure, previously calibrated in a pilot study^2^.

**Figure 1:**
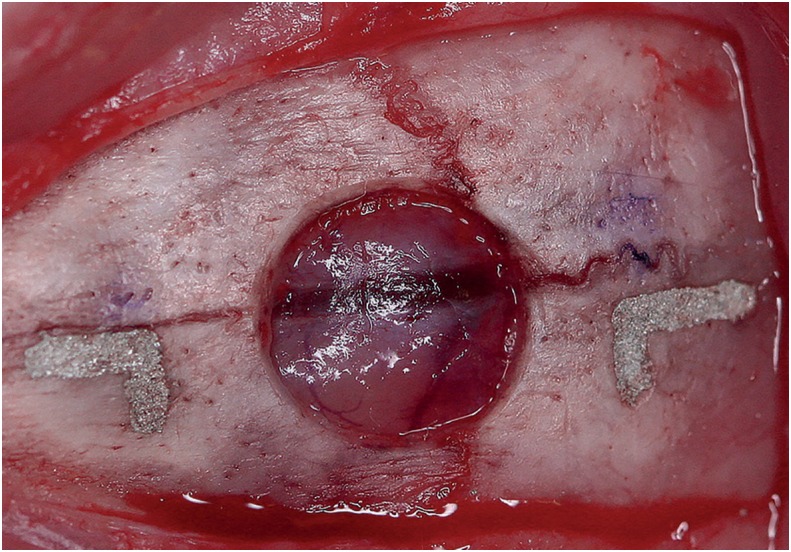
L-shaped marks conducted at 2 mm anteriorly and another 2 mm posteriorly to the margins of the critical size defect (CSD) with 5 mm diameter

For accomplishment of all experimental procedures, the rats were anesthetized with an intramuscular injection of xylazine (Vet Brands International Inc., Paulínia, SP, Brazil) (0.02 ml/kg) and ketamine hydrochloride (Vet Brands International Inc., Paulínia, SP, Brazil) (0.4 ml/kg). Trichotomy and antisepsis were performed on the dorsal cranium of each animal, then, a semilunar incision was performed on the calvaria and a full thickness flap was raised in posterior direction. A critical size defect (CSD) with 5 mm diameter was created with a trephine (JJGC Indústria e Comércio de Materiais Dentários S.A., Curitiba, PR, Brazil) at low speed, under abundant irrigation with sterile saline (Figure 1). Care was taken to avoid injury to the dura mater and brain during craniotomy^2^ ^,^ ^13^.

L-shaped marks were performed 2 mm anteriorly and 2 mm posteriorly to the surgical defect margins, using a carbide bur FG-700 (Microdont Micro Usinagem de Precisão Ltda., São Paulo, SP, Brazil) under continuous irrigation with sterile saline, which were then filled with amalgam (Figure 1). These indents allowed identifying the center of the original surgical defect during laboratory processing, as well as the location of original bone margins during histometric analysis^13^.

The surgical defect was filled only with blood clot in C group. In group PBMT, the defect was filled with blood clot and then irradiated with PBMT. Group AB was treated with autogenous bone removed from the cranial vault and ground. Group AB+PBMT was filled with autogenous bone followed by irradiation with PBMT. In groups treated with membrane, the defect was filled with blood clot (group M), autogenous bone associated with membrane (group M+AB) and autogenous bone irradiated with PBMT associated with membrane (M+AB+PBMT).

The flap was repositioned and sutured with silk suture 4-0 (Johnson & Johnson do Brasil Indústria e Comércio de Produtos para Saúde LTDA, São José dos Campos, SP, Brazil). Each animal received intramuscular injection of 24,000 units of penicillin G benzathine (Fort Dodge^®^ Saúde Animal Ltda., Campinas, SP, Brazil), and intramuscular dose of 3 mg/kg of ketoprofen (Laboratório Teuto Brasileiro S.A., Anápolis, GO, Brazil)^12^.

### Laser protocol

This study employed TheraLase DMC^®^, in continuous mode, infrared spectrum, with active medium GaAlAs, beam area of 0.028 cm^2^, wavelength of 808 nm, power of 100 mW, irradiance at target of 0.6 mW/ cm^2^, energy density of 210 J/cm^2^ *per* point during 60 s/point, applied on five points: four on the surface of the surgically created defect, according to clockwise positions (12 h, 3 h, 6 h, 9 h), besides a central point^2^. Each point received an energy dose of 6 J during 60 s/point, the area received a total energy of 30 J. Only one application was performed transoperatively.

In groups filled only with blood clot, the central point of application was the center of the blood clot, while for groups filled with autogenous bone the central point of laser application was determined after material placement. When the membrane was used, the central point of application was performed and, subsequently, the barrier was adapted^2^, the flap was repositioned and sutured.

### Tissue processing

The animals were killed after 30 days postoperatively, with 5 mg/ml of association of ketamine hydrochloride and xylazine. They were killed considering the phylogenetic scale, since, for the animals, this period corresponds to 90 days of healing in humans^18^.

The area of the original surgical defect and surrounding tissues were removed *en bloc,* the specimens were fixated in 10% neutral formalin, rinsed in tap water and decalcified in 18% ethylenediaminetetraacetic acid (Dinâmica^®^ Química Contemporânea Ltda., Diadema, SP, Brazil). After decalcification, each specimen was longitudinally divided into two blocks through the center of the original surgical defect, using the amalgam marks (L) as reference. The specimens were processed and embedded in paraffin. Subsequently, longitudinal serial sections with 6-Mm thickness were obtained, initiating from the center of the original surgical defect. The sections were stained with hematoxylin and eosin (H.E.).

The images of histologic sections were captured using a digital camera SPOT RT3 – 2540 Color Slider 2.0 Mp (SPOT ImagingSolutions, Diagnostic Instruments, Inc., Sterling Heights, EUA) connected to a microscope Olympus BX50 (Olympus Corporation, Shinjuko, Tokyo, Japan), at 2x magnification and saved in a computer.

### Histometric analysis

Histomorphometric analysis was conducted using the computer image analysis software ImageLab 2000 (Diracon Bio Informática Ltda., Vargem Grande do Sul, SP, Brazil) by an examiner blinded to the experimental groups and previously calibrated^2^ ^,^ ^6^.

For histological and histometric analyses, two histological sections were selected, representing the central area of the original surgical defect.

The area to be analyzed was delineated on each image and named total area (TA), corresponding to the calvaria bone region, where the defect was originally created. The area of newly formed bone (ANB) and the area of remaining particles (ARP), inside the TA, were also identified. The TA was measured in mm^2^ representing 100% of the area analyzed. The ANB and ARP were also measured in mm^2^ and calculated as percentages of TA^13^.

### Statistical analysis

Data were submitted to normality test (Shapiro- Wilk), which showed that they had normal distribution. All variables showed statistical differences between means according to the ANOVA parametric test (p<0.05). Multiple comparisons of ANB were submitted to the Tukey test (p<0.05). Analysis of the statistical test power was verified with a minimum power of 0.998.

## Results

During laboratory processing, 01 specimen in Groups C, AB, AB+M; 02 specimens in Groups M, AB+PBMT; and 03 specimens in Groups PBMT, PBMT+M were lost.

### Histological analysis

Variable bone extent was observed in all groups; however, no group exhibited complete closure of the surgical defect. The groups did not show intense inflammatory infiltrate.

**Figure 2:**
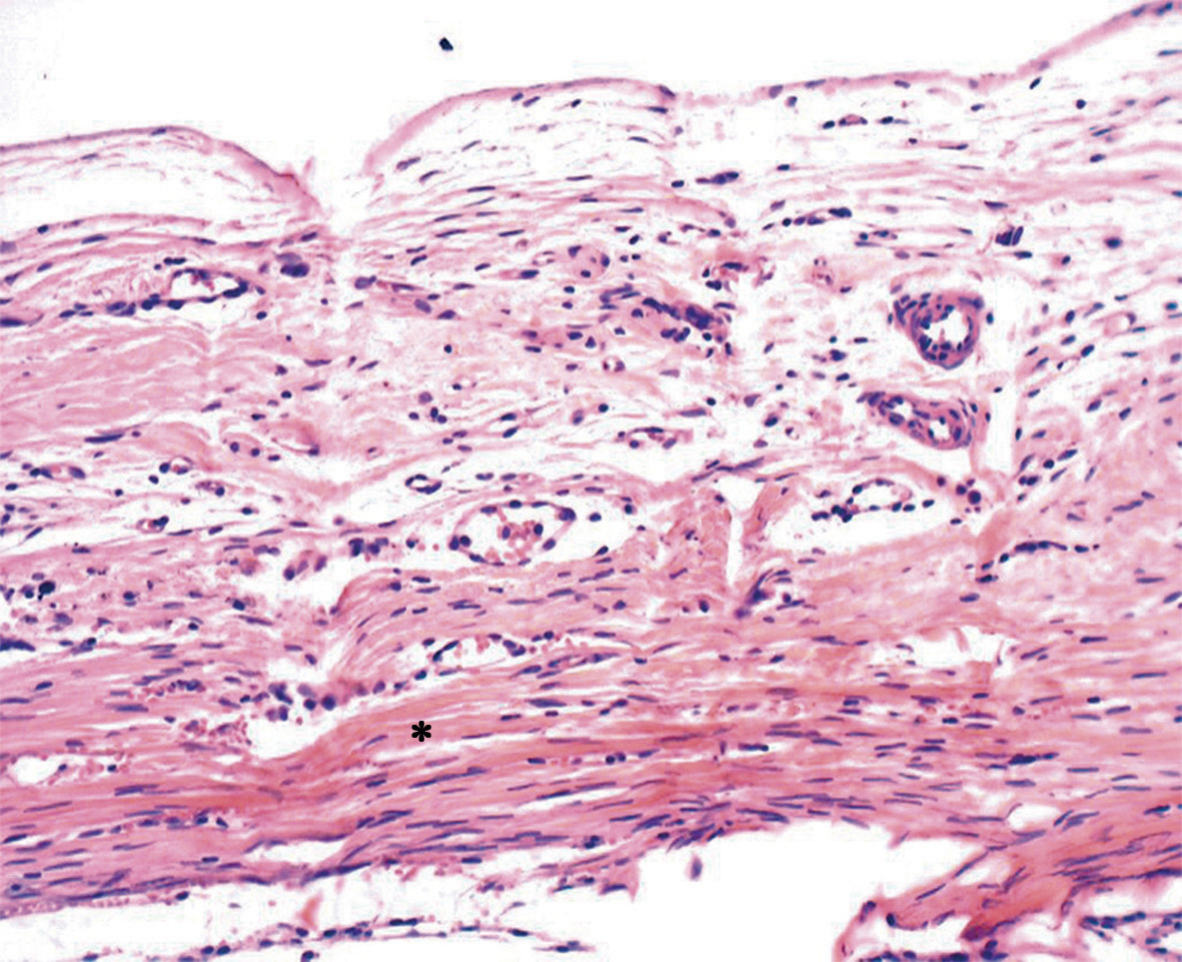
Group C: Presence of loose connective tissue with parallel collagen fibers and few inflammatory cells (*) (40x)

Group C - In these specimens, the extent of the surgical wound was almost entirely filled with loose connective tissue and parallel collagen fibers. There was a small area of new bone restricted to the defect margins, thus not maintaining the original calvaria thickness (Figure 2).

**Figure 3:**
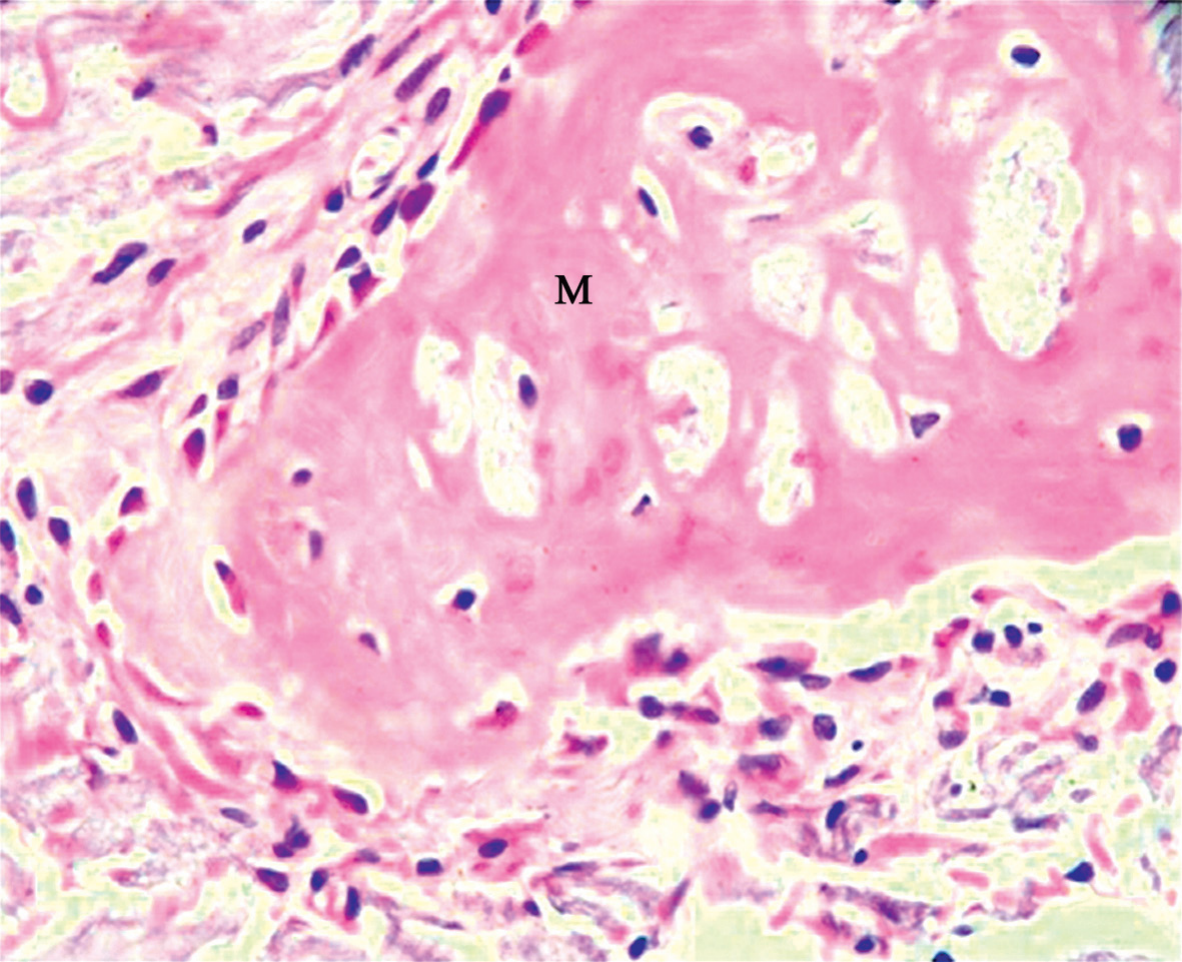
Group M: Membrane with bone formation inside it (M) (40x)

Group M - This group exhibited well-organized connective tissue, with parallel collagen fibers, ossification in variable extents of the membrane with bone formation above it (Figure 3) and beyond the limits of the original defect, yet the calvaria thickness was not maintained.

**Figure 4:**
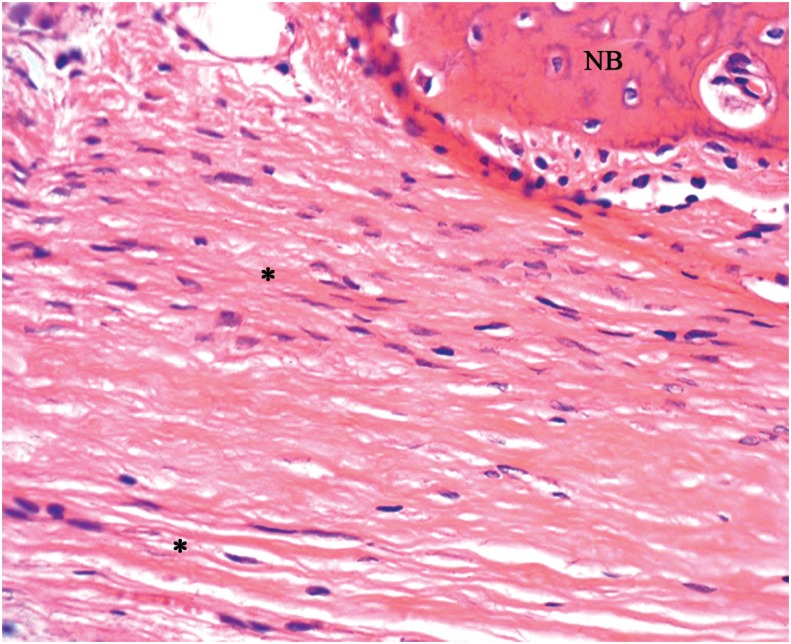
Group PBMT: Dense connective tissue, with parallel fibers (*) and newly formed bone tissue (NB) (40x)

Group PBMT - The connective tissue was well organized, with parallel collagen fibers, more mature bone tissue extending toward the center of the defect in some specimens (Figure 4), yet the original calvaria thickness was not maintained.

**Figure 5:**
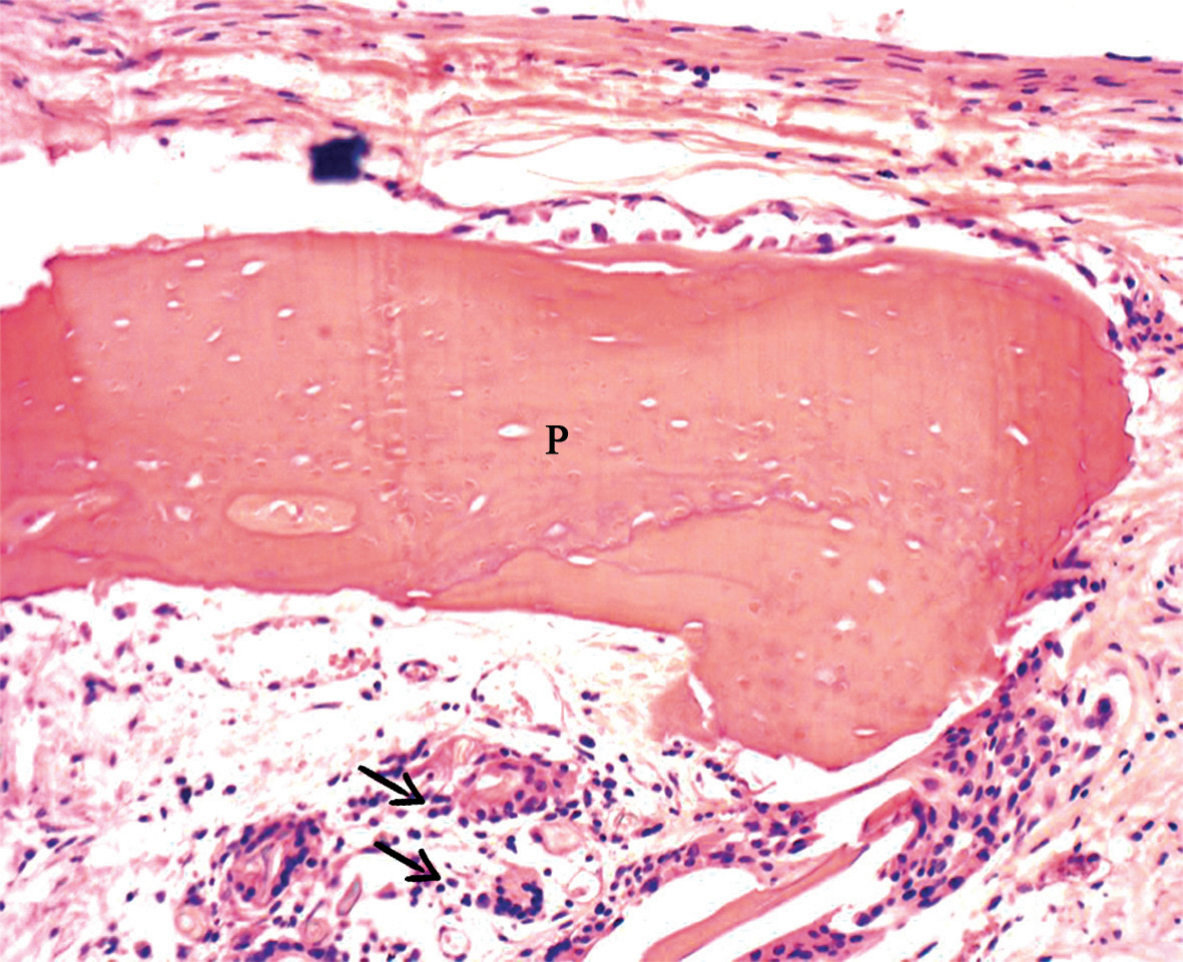
Group AB+PBMT: Presence of particle (P) in central surgical defect and some giant cells (arrows) (20x)

Group AB - This group showed well-organized connective tissue, with presence of osteoid matrix and fibroblasts in the defect. New bone was observed in variable extents of the defect, with few remaining particles (Figure 5).

**Figure 6:**
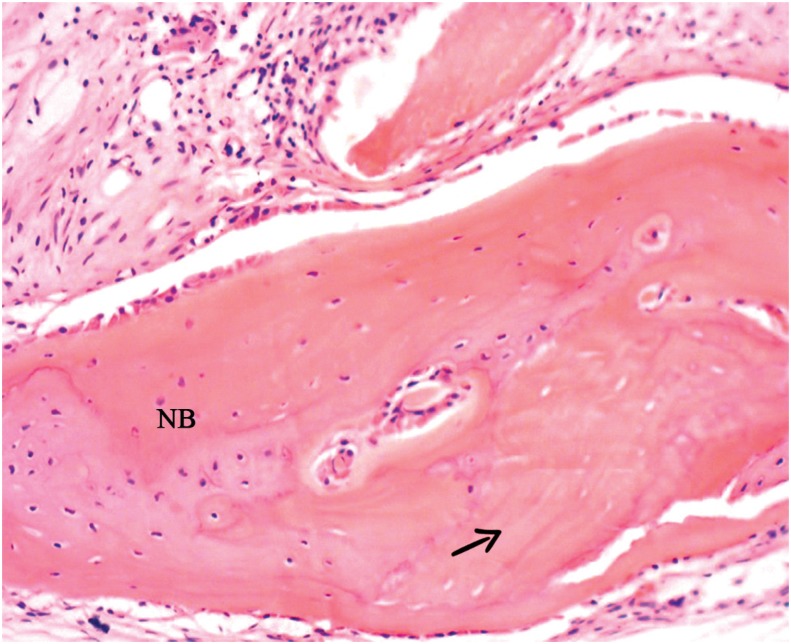
Group AB+PBMT: Presence of newly formed bone (NB) surrounding particle of autogenous bone (arrow) (40x)

Group AB+PBMT - There was new bone formation in variable extents, yet with few remaining particles (Figure 6).

**Figure 7:**
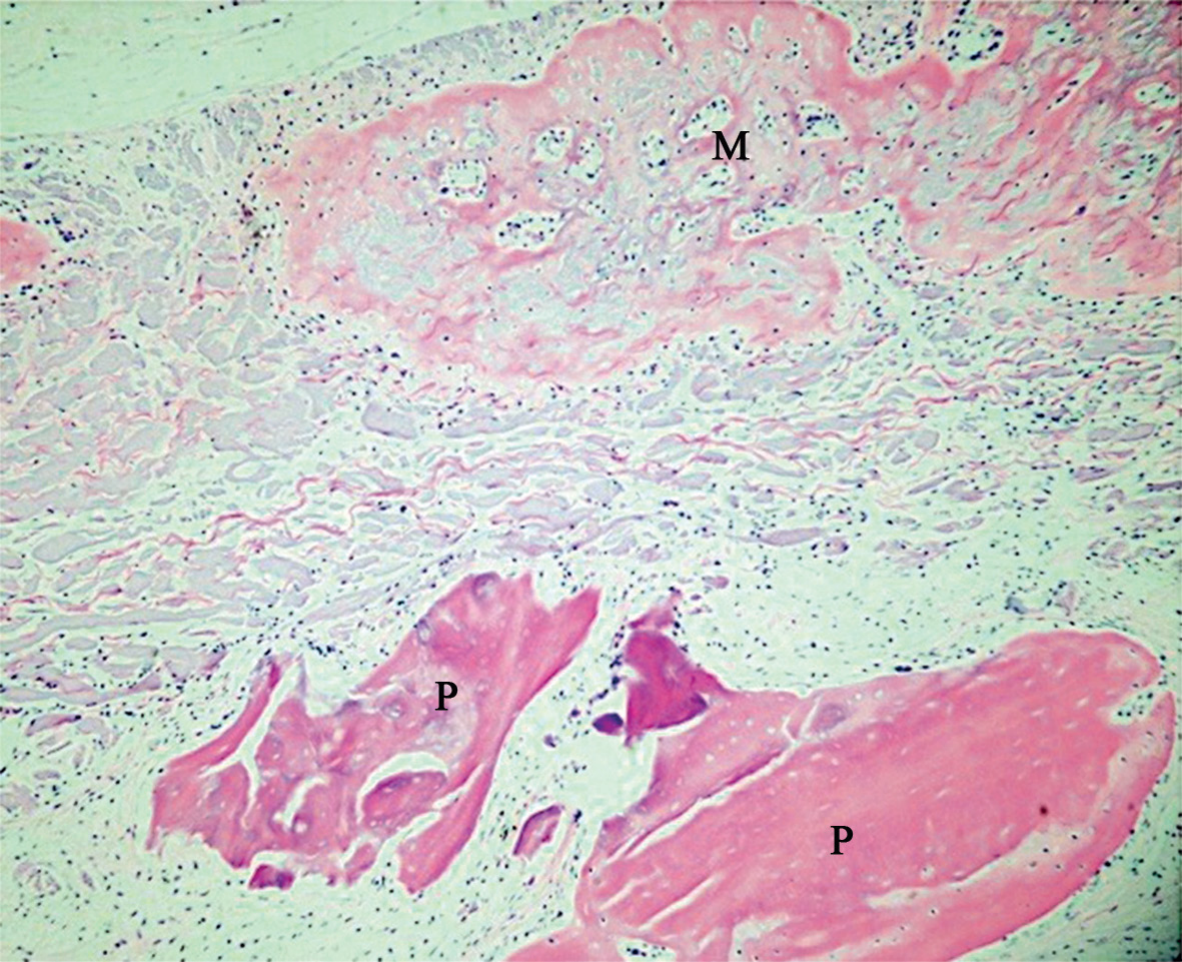
Group AB+M: Central region of surgical defect with new bone formation at the membrane region (M), and some particles of autogenous bone (P) (20x)

Group AB+M - Similarly, there was ossification of membrane with bone formation above it (Figure 7). Remaining particles of autogenous bone were observed at the center of the defect, yet with osteoid matrix around it.

**Figure 8:**
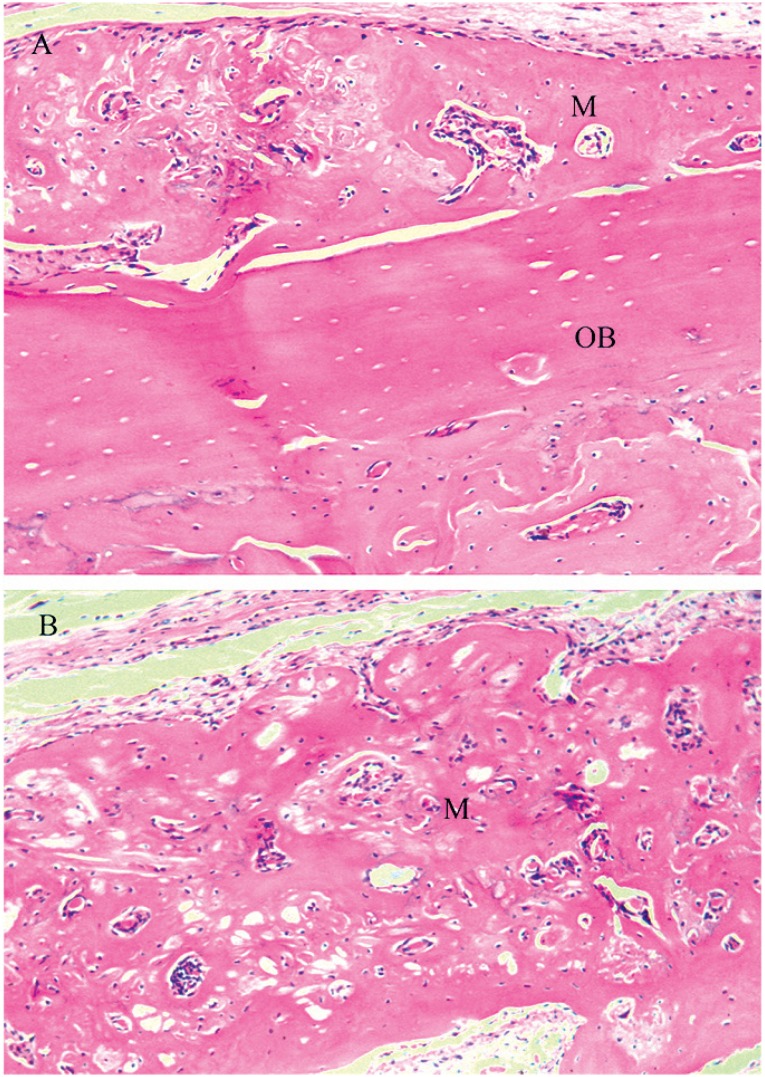
Group PBMT+M: A - Bone formation inside the membrane, membrane ossified (M), original bone (OB). B - Bone formation in the center of the defect (40x), ossified membrane (M)

Group PBMT+M - This group exhibited variable extent of bone tissue inside the defect, yet without complete closure. Ossification of the membrane was observed (Figure 8), with formation of bone tissue above it, often surpassing the limits of the original calvaria, yet without maintenance of thickness. The connective tissue was well organized, with parallel collagen fibers and great quantity of fibroblasts.

**Figure 9:**
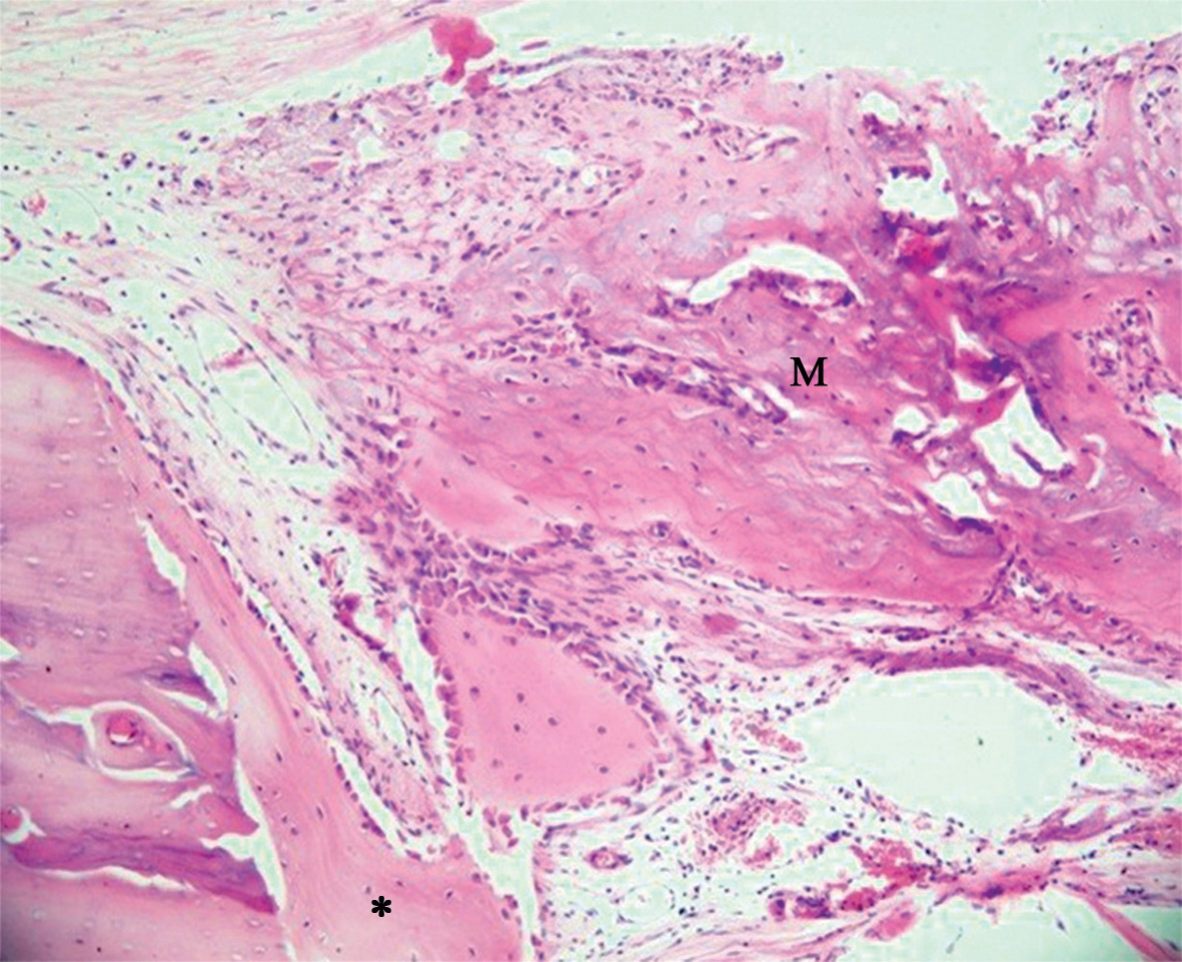
Group AB+PBMT+M: Bone formation at the region of the membrane (M) and margin of the surgical defect (*) (40x)

Group AB+PBMT + M - This group exhibited presence of remaining particles with osteoid matrix around it, yet the quantity of ANB was limited. The membrane was ossified in several specimens (Figure 9) with new bone formation beyond the limits of the original calvaria, yet without maintenance of thickness.

### Histometric and statistical analysis

Table 1 shows the descriptive analysis of ANB for all groups and comparison between them.

**Table 1:** Means, standard deviations (SD), Q25, medians and Q75 values of area of newly formed bone in % (ANB), and comparison between groups (Tukey test)

Groups	N	Means	SD	Q25	Medians	Q75
C^a^	9	9.96	4.49	6.64	9.52	14.32
M^b,d,e,g,h^	8	47. 43	15.73	35.6	39.26	63.74
PBMT^b,c,d,e,g,h^	7	47.67	8.66	43.96	44.58	55.41
AB^d,h^	9	25.1	16.59	19.36	25.10	36.83
AB+PBMT^d,e^	8	39.15	16.72	26.89	40.41	53.32
AB+M^a,d,e,f,h^	9	22.72	13.83	19.24	22.72	37.73
PBMT+M^g^	7	64.09	7.62	25.08	64.09	74.53
AB+PBMT+M^e,h^	10	35.82	7.68	25.12	35.82	42.00

* Same letters represent no statistical difference (significance level of 5%).

* Strength of the statistical test=99.8%, considering an alpha of 5%

When the control group (9.96±4.49%) was compared with the others, statistically significant differences in ANB were observed in all groups, except for group AB+M (22.72±13.83%).

Comparison of group PBMT (47.67±8.66%) with the other groups showed statistically significant differences in ANB only for group AB+M (22.72±13.83%).

Multiple comparisons of ANB values between groups treated with collagen membrane and the others showed statistically significant difference only for group M (47.43±15.73%) with AB+M (22.72±13.83%) in the evaluation of ANB.

Statistically significant difference was found in multiple comparisons of ANB values of group PBMT+M (64.09±7.62%) with the other groups (AB+M – 22.72±13.83%; AB+PBMT+M – 35.82±7.68; AB – 25.1±16.59 and AB+PBMT – 39.15±16.72).

Analysis of ANB between group AB+M and the other groups did not show statistically significant differences between them.

Comparison of group AB+PBMT+M with groups AB and AB+PBMT did not show statistically significant differences, as well as comparison between groups AB and AB+PBMT, when the area of new bone was analyzed.

The ARP means were calculated in percentage. Similar quantities of remaining particles were observed for groups AB+M (14.93±8.92%) and AB+PBMT+M (14.76±6.58%).

## Discussion

The study showed that PBMT may be effective for bone repair when associated with bone regeneration techniques, rejecting the null hypothesis.

The bone tissue has wide ability to regenerate, however there are limits for spontaneous repair. In case of very wide defects, in which the vascularization capacity is impaired or there is mechanical instability, the ability to recover the structure and function is limited. Therefore, several techniques and materials have been investigated to accelerate bone repair, and low-level laser has been a therapeutic option often indicated for that purpose^2^ ^−^ ^4^ ^,^ ^6^ ^,^ ^17^ ^,^ ^20^ ^,^ ^25^. Nonetheless, there are controversies as to its applicability, since the success of this therapy depends not only on the dose, but also on the time and irradiation mode^3^ ^,^ ^5^.

The PBMT is accessible, requires no coadjutant drugs for application and causes no thermal effects to irradiated tissues^26^.

Our study comprised a single application of low-level laser with λ 808 nm, power of 100 mW, energy density of 210 J/cm^2^ per point for 60 s, on 5 points on the CSD^2^ ^,^ ^6^. This method was employed because previous studies showed that PBMT has greater effect on cells if applied on the first stages of repair, when there is greater cell proliferation and division, promoting increased volume of newly formed bone^2^ ^,^ ^3^ ^,^ ^16^ ^,^ ^26^ ^,^ ^27^. After 15 days, the cells are in the differentiation stage, thus laser application after this period would not be effective^23^. Similarly, killing the animals after a period longer than 30 days would not influence on the outcomes, since the effect of laser application occurred at the initial stages of bone repair, corroborating the report of Weber, et al.^27^ (2006), who observed bone formation at 30 days postoperatively.

Thus, doubts still remain on whether the effects of laser application in several healing stages may have better outcomes if compared to a single application with ideal dose transoperatively.

Different from other studies, our group demonstrated that a single session with ideal dose and wavelength was effective to accelerate bone repair, since the margins of the defect are irradiated on four points, besides application on the central region of the defect, determined after filling with the biomaterial^2^ ^,^ ^6^. Our results differ from previous investigations stating that a single laser application may not be effective, since the light may not reach the margins of the surgical defect, and consequently, the cells at that region would not be stimulated^26^. Also, the accomplishment of several sessions of laser application postoperatively may delay the treatment and be financially unfeasible for the patient, precluding the utilization of this technique in clinical practice.

In our study, the area analysis of newly formed bone demonstrated that PBMT accelerated bone repair in all irradiated groups. In these groups, connective tissue with well-organized collagen fibers were observed, as well as greater bone tissue formation, indicating that the therapy accelerated the repair process, corroborating the results of previous studies^4^ ^,^ ^10^ ^,^ ^17^ ^,^ ^25^. This may have occurred because laser rises the ATP levels^23^, increases the osteoblastic activity, stimulates angiogenesis, reduces the osteoclastic activity^15^, promotes the organization of collagen fibers, provides proliferation of epithelial cells and fibroblasts, enhancing the collagen synthesis^1^ and increasing the expression of genes related with inflammation and angiogenesis^25^.

The GaAlAs laser used in this study has wavelength outside the light spectrum (λ 808 nm), between infrared and regions close to red, promoting ideal penetration into the bone tissue^22^ ^,^ ^25^, increasing the proliferation of osteoblastic cells, collagen matrix and new bone formation^2^ ^,^ ^6^ ^,^ ^20^ ^,^ ^25^.

In groups treated with PBMT, bone formation was significantly higher than in group C. When PBMT was applied in isolation, ANB analysis demonstrated good outcomes compared to non-irradiated groups, which demonstrates that utilization of this protocol *per se* was effective to accelerate bone repair.

The results were better when therapy with PBMT was associated with utilization of collagen membrane (group PBMT+M), achieving the best ANB outcome. This may be explained because, in guided bone regeneration, space maintenance by utilization of membranes is a prerequisite for treatment success^14^. Also, its utilization may avoid graft resorption and assure stability of the graft material. This may explain the fact that, when group M was compared to the others, it showed statistically significant differences in relation to groups C and AB+M. We did not find any study in the literature similar to our research, which uses collagen membrane associated with autogenous graft and/or low-level laser.

Even though no significant differences were observed between groups, group AB+PBMT+M exhibited greater quantity of new bone formation than AB+M. In these groups, when the membrane was associated with autogenous graft and/or laser, there was new bone formation below, above and inside the collagen membrane; however, the ANB observed beyond that the TA was not considered in this study due to the methodology employed, which may have reduced the calculated percentage of new bone in each specimen in the study groups.

The same finding may have occurred for the group AB+PBMT+M compared to groups AB+PBMT; PBMT+M; M and PBMT, since the histometric analysis did not show greater quantity of ANB, different than the observed on histological analysis. The potential for ossification and calcification of collagen matrices close to bone tissue, where the density of surrounding bone may suggest increased ossification of the membrane and bone formation around it^28^, is one characteristic of the collagen membrane, besides the low antigenicity^28^, high biocompatibility^19^ ^,^ ^28^ and low cytotoxicity^19^ ^,^ ^28^.

Autogenous bone is the material of choice for bone regeneration due to its osteoinductive properties, thus it was used as positive control in this study, although its association with PBMT promoted no additional bone regeneration, presumably due to the applied dose or even interference with the production of growth factors. Weber, et al.^27^ (2006) evaluated the effect of GaAlAs laser associated with autogenous grafts in rat calvaria and achieved positive outcomes in transoperative applications, revealing greater quantity and quality of bone. Also, in addition to greater bone resorption, the quantitative and qualitative analyses of new bone in their study were also greater when the surgical site was irradiated. When laser was applied only on the graft, at 30 days, despite presence of remaining particles of the grafted material inside the cavity, there was no induction of new bone formation.

In our study, both the receptor site and graft were irradiated, and even though no statistically significant differences were observed between groups AB and AB+PBMT, we believe that the laser was able to accelerate the bone repair, since histological analysis showed new bone formation toward the center of the defect, as well as presence of osteoid matrix around the remaining particles in some specimens, demonstrating that laser application at the initial stages of repair may induce osteogenesis and aid the integration of the grafted material with the receptor site^17^ ^,^ ^23^ ^,^ ^27^.

Several studies in the literature demonstrate that filling of bone defects only with blood clot is not sufficient to maintain the bone architecture^21^. To reduce this volume loss, studies demonstrated the possibility to fill bone defects by tissue regeneration techniques with or without bone substitutes^2^ ^,^ ^6^ ^,^ ^21^, corroborating our findings, in which group C showed worse ANB results, with loss of the original calvaria thickness.

Concerning the quantity of remaining particles, groups M+AB and M+AB+PBMT showed similar ARP quantities ARP after 30 days, with variable quantity of new bone formation. Our findings differ from those observed in the study of Weber et al.^27^ (2006), at the same timing of analysis, in which no signs of bone formation were observed despite the presence of remaining particles. Groups AB and AB+PBMT showed no statistically significant quantities of remaining particles. Therefore, when PBMT is used as a coadjutant treatment or even in isolation, it may aid to improve the results of new bone formation in critical size defects^17^ ^,^ ^20^.

Future studies should be conducted using advanced techniques for analysis, e.g. immunohistochemistry and microtomography, to evaluate the effect of low- level laser on the initial stages of bone repair, to complement the histomorphometric analysis.

## Conclusion

Considering our results, the PBMT utilization may be effective for bone repair when associated with bone regeneration techniques. Thus, the null hypothesis was rejected.
